# Structural basis of outer membrane biogenesis and cell division by Tol/Pal nanomachinery

**DOI:** 10.1126/sciadv.adw6719

**Published:** 2026-03-06

**Authors:** Yatian Chen, Biao Yang, Ruixin Fan, Xiaofeng Duan, Zhizhen Jin, Danyang Li, Xiangning Li, Zhengyu Zhang, Changjiang Dong

**Affiliations:** ^1^ Department of Thyroid and Breast Surgery, Zhongnan Hospital of Wuhan University, School of Pharmaceutical Sciences, Wuhan University, Wuhan 430071, China.; ^2^ Key Laboratory of Combinatorial Biosynthesis and Drug Discovery, Ministry of Education, School of Pharmaceutical Sciences, Wuhan University, Wuhan 430071, China.; ^3^ State Key Laboratory of Oral & Maxillofacial Reconstruction and Regeneration, Key Laboratory of Oral Biomedicine Ministry of Education, Hubei Key Laboratory of Stomatology, School & Hospital of Stomatology, Wuhan University, Wuhan 430079, China.; ^4^ Cryo-EM Center, Core facility of Wuhan University, Wuhan University, Wuhan 430071, China.

## Abstract

TolQRA, a key member of the proton motive force (PMF) family including MotAB and ExbBD, transduces PMF from the inner membrane to the outer bacterial envelope. This mechanism compensates for the absence of conventional energy sources in the outer membrane compartment of Gram-negative bacteria. Here, we present cryo–electron microscopy structures of the TolQRA complex at pH 5.4 and pH 8.0, resolved at 3.18 and 3.60 angstroms, respectively. Our findings revealed that TolQRA has a stoichiometry of 5:2:5, with key residues mediating interactions between TolQ, TolR, and TolA. Notably, the nanomachine has appeared to exhibit an asymmetric arrangement, which may be consistent with a two-gate mechanism for proton translocation and energy transfer. These insights illuminate the mechanism of energy transduction in TolQRA, offering parallels with the ExbBD-TonB and MotAB systems. Furthermore, this work provides a foundation for the development of innovative therapeutics that target the critical TolQRA complex.

## Teaser

Cryo-EM structures of the TolQRA complex at pH 5.4 and pH 8.0 were resolved at 3.18 and 3.60 Å, with the stoichiometry of 5:2:5.

## INTRODUCTION

Gram-negative bacteria have two membranes, the outer membrane (OM) and the inner membrane (IM), with a peptidoglycan layer in the aqueous periplasm between the OM and the IM (*1*). The OM plays essential roles in nutrient import, biofilm formation, bacterial movement, pathogenesis, drug resistance, and survival in different environments (*2*). However, the periplasm does not contain adenosine triphosphate and the OM lacks a proton motive force (PMF), which poses a great challenge for the OM to carry out these functions (*2*, *3*). A hetero-oligomeric nanomachine, TolQRA, spans the periplasm of Gram-negative bacteria to interact with the periplasmic protein TolB and the OM protein Pal. These five proteins form the Tol-Pal system, which was originally identified as a colicin import–associated protein (*4*–*6*) and was subsequently found to be related to the translocation of filamentous bacteriophages into the cytoplasm (*7*–*9*). Studies have confirmed that the Tol-Pal system plays a critical role in OM invagination during bacterial cell division (*10*–*13*), OM biogenesis (*13*–*15*), pathogenesis, and drug resistance (*15*–*17*) and is an important target for the development of drugs and vaccines (*18*).

**Fig. 1.**
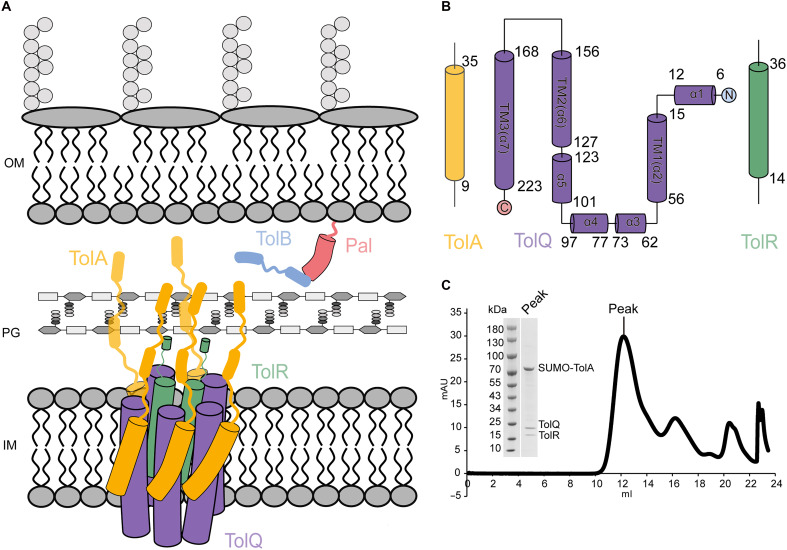
Schematic diagram of TolQRA. (**A**) Schematic representation of the Tol-Pal system. (**B**) A topology diagram of the secondary structure of TolA, TolQ, and TolR determined by cryo-EM in this study. TolQ (purple), TolR (green), TolA (orange), TolB (blue), and Pal (red) are shown as cylinders. (**C**) Size-exclusion chromatography and SDS–polyacrylamide gel electrophoresis of purified TolQRA.

Most Gram-negative bacteria have a Tol-Pal system (*19*). The Tol-Pal system transduces the PMF energy generated by the inward transport of protons across the IM to both the OM and peptidoglycan layers by TolB-Pal. In *Escherichia coli*, TolQ, TolR, and TolA form a protein nanomachinery in the IM. TolQ contains 230 residues consisting of three transmembrane (TM) helices, and TolR has 141 amino acids that form an N-terminal TM helix and a C-terminal domain, whereas TolA has 421 residues divided into an N-terminal TM helix, a linker domain containing two helices, and a globular C-terminal domain (Fig. 1A) (*20*, *21*). Pal is a 173-residue lipoprotein comprising an N-terminal loop and a C-terminal structural domain, anchored to the OM via an N-terminal lipid moiety that is critical for its function (Fig. 1A) (*10*, *22*). TolB is a soluble periplasmic protein that interacts with Pal to form a complex (Fig. 1A). Overall, these two machineries traverse the IM, periplasm, and OM to transduce the energy required for critical and fundamental biological processes (*23*). Crystal structures of TolB and the Pal complex (*24*, *25*), TolB in complex with a peptide of the colicin E9 (*26*, *27*), the TolA C-terminal domain in complex with a colicin A N-terminal peptide (*28*), and a nuclear magnetic resonance structure of the TolA C-terminal domain bound to a TolB N-terminal peptide (*10*), as well as a cryo–electron microscopy (cryo-EM) structure of the colicin E9 OM translocon (*29*), have been reported. These structures reveal that TolB can bind Pal, colicin E9, and TolA, whereas TolA can bind TolB and colicin A. Cell biological studies have shown that the Tol-Pal system uses the PMF to transport Pal and TolQ to the septum (*10*–*12*). Recently, a cryo-EM structure of TolQR at a resolution of 4.3 Å was reported (*30*), revealing that the stoichiometry of TolQ and TolR is 5:2. However, the Tol-Pal molecular mechanism currently proposed is based mainly on studies of systems homologous to TolQR, such as ExbBD (*31*, *32*) and MotAB (*33*), which propose models for the stator and rotor. Both ExbBD and MotAB have molecular stoichiometries of 5 (ExbB/MotA) and 2 (ExbD/MotB) and use PMF to actively import nutrients and drive flagellar rotation (*34*–*41*), respectively. Despite notable achievements in understanding the Tol-Pal system, the mechanism by which TolQR transduces PMF from the IM through TolA remains poorly understood.

Here, we report the cryo-EM structures of the *E. coli* TolA, TolQ, and TolR protein complex at pH 5.4 and pH 8.0 at resolutions of 3.18 and 3.60 Å, respectively, mimicking the TolQRA machinery in the protonated and the resting states (*36*, *42*). Combined with mutagenesis, functional assays and particle three-dimensional (3D) variability analysis, we tentatively propose that protons may promote TolQRA to transduce energy to the OM via a putative alternating open-and-closed mechanism involving two gates. Our findings offer a framework for understanding how the Tol-Pal system regulates bacterial cell division, OM biogenesis, and drug resistance and offer insight into the function and mechanism of the TolQRA-related ExbBD-TonB and MotAB machineries.

## RESULTS

### TolQRA adopts a unique 5:2:5 stoichiometry

**Fig. 2.**
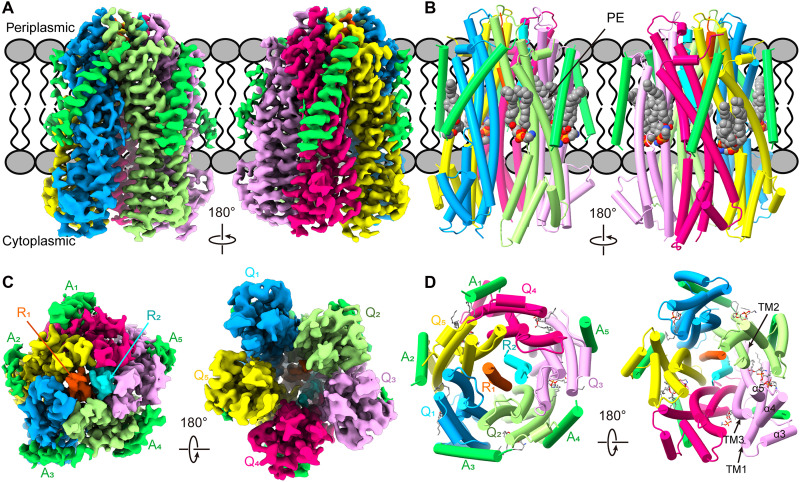
Cryo-EM structure of TolQRA (pH 5.4). (**A**) Cryo-EM map of TolQRA (pH 5.4), viewed from front and back. TolQ_1_ (sky blue), TolQ_2_ (limon), TolQ_3_ (pink), TolQ_4_ (hot pink), and TolQ_5_ (yellow) are shown in a surface representation for density, and TolR_1_ (orange) and TolR_2_ (cyan) are also shown. The five copies of TolA are colored in green. (**B**) Cartoon representation of the TolQRA (pH 5.4) complex in front and back views. (**C**) Top view and bottom view of the TolQRA (pH 5.4) cryo-EM map. (**D**) Cartoon representation of the TolQRA (pH 5.4) complex in the top and bottom views.

The *E. coli* genes encoding TolQ, TolR, and TolA were subsequently cloned and inserted into a pTrc99a plasmid with an 8-His-tag on the N terminus of TolA (Materials and Methods). TolQ, TolR, and TolA were coexpressed and purified via nickel affinity and size-exclusion chromatography (Materials and Methods), which confirmed that TolQ, TolR, and TolA formed a stable complex (Fig. 1, B and C). Previous structural studies of ExbBD have demonstrated that it exists in a resting state at pH 7.5 and a protonated state at pH 4.5 (*36*, *42*). To characterize the dual structural states of TolQRA while accounting for protein stability, we determined the cryo-EM structures of the TolQRA complex at pH 8.0 and pH 5.4 in this study. The TolQRA machinery was concentrated to 6 mg/ml in buffer A supplemented with 0.003% lauryl maltose neopentyl glycol (LMNG) for cryo-EM structure determination at pH 8.0 (Materials and Methods). To determine the TolQRA structure at pH 5.4, the size exclusion chromatography buffer was switched to buffer E supplemented with 0.003% LMNG (Materials and Methods). The cryo-EM structures of TolQRA at pH 5.4 and pH 8.0 were determined to be 3.18 and 3.60 Å (Fig. 2, A and B, and figs. S1 and S2), respectively. The cryo-EM densities are suitably high resolution for us to unambiguously construct near-atomic models, in which TolQ residues Met6–Phe224, TolR residues Lys13–Tyr35, and TolA residues Gly8–Asn38 were built in the models (Fig. 2, A to D), and the densities of the remaining residues were invisible, suggesting that these residues are disordered or more dynamic (fig. S3). As the TolQRA pH 5.4 structure showed better overall resolution, the TolQRA pH 5.4 structure was used unless otherwise stated.

There are five copies of TolQ, two copies of TolR, and five copies of TolA in the structures, suggesting that the TolQRA machinery has a 5:2:5 stoichiometry (Fig. 2D). To our knowledge, this study reveals TolQRA’s stoichiometry, and the exact stoichiometry of the homolog system ExbBD-TonB is still unclear (*31*, *35*). TolQ forms a pentameric α barrel in the IM, and TolR adopts a dimer and plugs inside TolQ from the periplasm, and five TolA molecules attach to the outside of TolQ in the IM (Fig. 2, A and B).

### One TolA protein interacts with two neighboring TolQ protomers

The cryo-EM structures revealed that the TolQ protomer consists of seven α helices. Two of these helices, α2 and α6, traverse the IM (TM helices 1 and 2) and extend into the cytoplasm. In contrast, α7 crosses the IM (TM helix 3) and extends into the periplasm. The α1 helix, an accessory helix, is located in the periplasm, whereas accessory helices 2, 3, and 4 (α3, α4, and α5) reside in the cytoplasm (Fig. 1B). The TM domains are not oriented perpendicular to the membrane but rather tilt approximately 60° from the left to the right relative to the membrane. In contrast, the cytoplasmic domain exhibits a right-to-left turn. TolQ forms a pentamer, with protomers labeled Q_1_ to Q_5_, through extensive interactions between neighboring TM and cytoplasmic domains (Figs. 2D). The buried surface areas between adjacent protomers vary, suggesting conformational differences: Q_1_-Q_2_ (1241.4 Å^2^), Q_2_-Q_3_ (912.2 Å^2^), Q_3_-Q_4_ (1316.4 Å^2^), Q_4_-Q_5_ (1438.5 Å^2^), and Q_5_-Q_1_ (1123.7 Å^2^). The largest gap occurs between Q_2_ and Q_3_, whereas the narrowest gap is observed between Q_4_ and Q_5_ (fig. S4A).

Electrostatic analysis revealed a highly charged periplasmic surface with both negative and positive regions. In contrast, the cytoplasmic surface is characterized by distinct positively and negatively charged belts (fig. S4, B and C). Superimposition of Q_2_, Q_3_, Q_4_, and Q_5_ onto Q_1_ revealed high structural similarity, with root mean square deviation (RMSD) values ranging from 1.00 to 1.26 Å over 215 aligned residues. The main conformational changes occur in accessory helices 1, 2, 3, and 4, as well as the periplasmic domains of α2 (TM1), α6 (TM2), and α7 (TM3) (fig. S4D).

**Fig. 3.**
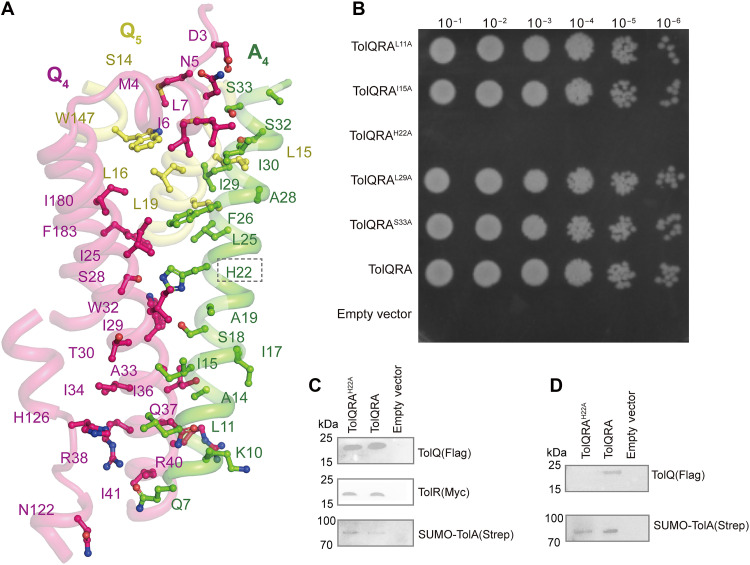
Residues that interact with TolA and TolQ. (**A**) Interactions between TolA_4_ (green), TolQ_4_ (hot pink), and TolQ_5_ (yellow). The side chains are shown as ball-and-stick. (**B**) Functional assay of TolQRA mutants. The mutant TolQRA^H22A^ caused bacterial death. All assays were performed in LB medium supplemented with rifampicin (2.5 μg/ml). (**C**) Western blot showing the expression levels of TolQ, TolR, and TolA in the TolQRA^H22A^ mutant and wild type. (**D**) Western blot results of the TolA^H22A^ mutant pull-down assay. The TolA^H22A^ failed to pull down TolQ.

There are five densities surrounding the TolQ TM domains, which correspond to the TM helices of TolA, labeled A_1_-A_5_ on the basis of their primary TolQ TM domain contact (Fig. 2D). While the densities for A_1_ and A_4_ are the most pronounced, those for A_2_ and A_3_ are also discernible, whereas the density for A_5_ is less defined, suggesting greater mobility (fig. S3). TolA crosses the IM at an angle of approximately 60° to the membrane and interacts extensively with two neighboring TolQ protomers (Fig. 3A). TolA residues Gln7, Lys10, Leu11, Ala14, Ile15, Ile17, Ser18, Ala19, His22, Leu25, Phe26, Ala28, Leu29, Ile30, Ser32, and Ser33 form hydrophobic interactions with the TM domain of TolQ; protomer Q_4_ TM1 (Ile25, Ser28, Ile29, Thr30, Ala33, Ile36, Gln37, Arg38, Arg40, and Ile41); protomer Q_4_ TM3 (Ile180, Phe183); protomer Q_5_ TM2 (Trp147); and protomer Q_5_ TM1 (Leu15, Leu16, Leu19) (Fig. 3A). To test whether these interactions are critical for TolQRA functionality, we generated a *tolQRA* deletion strain named WYD1 and TolA mutants of Leu11→Ala (L11A), Ile15→Ala (I15A), His22→Ala (H22A), Leu29→Ala (L29A), and Ser33→Ala (S33A) and performed functional assays in the presence of rifampicin. Previous experiments have shown that the absence of Tol-Pal proteins in Salmonella impairs its resistance to rifampicin (*14*). Our experimental results similarly show that WYD1 displays severe growth impairments in the presence of rifampicin. All mutants and complementation plasmid were functionally validated in WYD1, with normal expression of TolQ and TolR confirmed. Our assays revealed that the mutant TolA mutant H22A caused bacterial death, but the TolA mutants L11A, I15A, L29A, and S33A did not affect cell growth (Fig. 3, B and C). Previous studies have reported that the assembly of the TolQR complex with TolA is facilitated by a conserved SHLS motif located within the TM structural domain of TolA (*43*–*45*). Mutation of the H22 residue in the TolA SHLS motif markedly disrupts the interaction between TolA and the TolQ-TolR motor complex, thereby inhibiting the energy-dependent conformational change of TolA and its interaction with Pal in the OM (*30*, *43*–*47*). Our pull-down assay showed that when pulldown of TolQ was performed using TolA, TolQ could be detected in the wild-type sample but not in the H22A mutant. Consistent with previous studies, this confirms that that H22 is involved in the interaction between TolA and TolQ, corroborating that the TolA-TolQ interaction is essential for TolQRA function (Fig. 3, B to D).

### TolR dimerization and unequal positioning in the TolQ pentamer

**Fig. 4.**
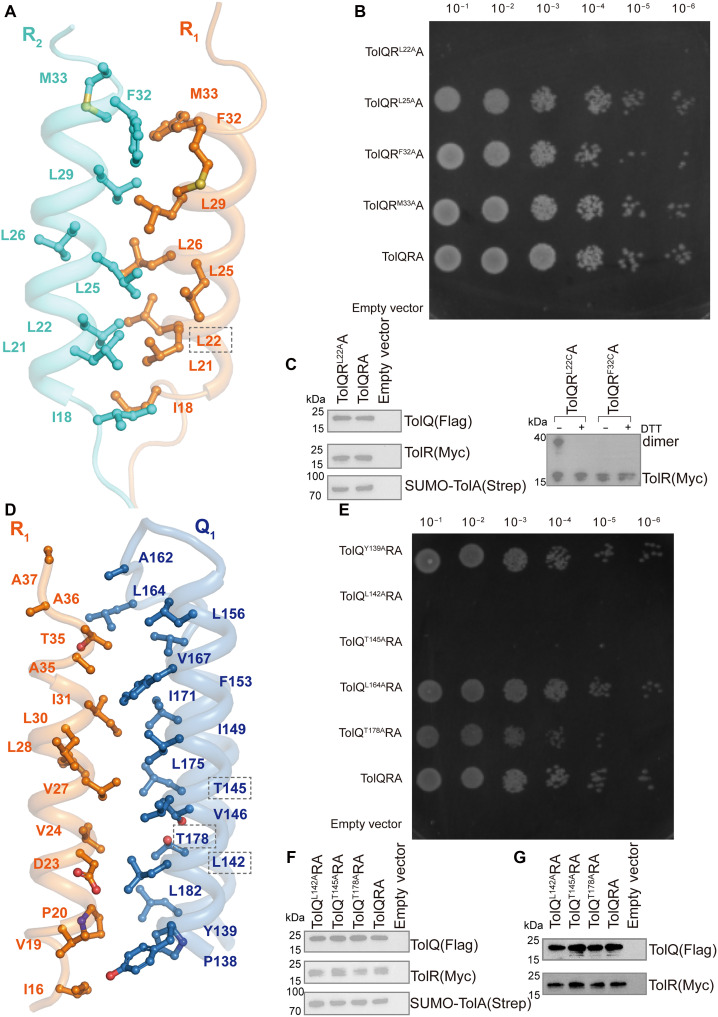
TolR residues involved in TolR dimerization and interaction with TolQ. (**A**) The interacting residues between TolR_1_ (orange) and TolR_2_ (cyan) in the TolR dimer. The side chains of the residues are shown in ball-and-stick representation. (**B**) Functional assay of TolR mutants, where the TolQR^L22A^A mutant exhibited severe cell growth defects. All assays were performed in LB medium supplemented with rifampicin (2.5 μg/ml). (**C**) Western blotting analysis revealed the expression levels of TolQ, TolR, and TolR in the TolQRA mutants, as well as disulfide bond cross-linking in TolR. The TolR^L22C^ mutant formed a disulfide bond, whereas the TolR^F32C^ did not. (**D**) The interaction residues between TolR_1_ (orange) and TolQ_1_ (sky blue), with the side chains displayed in ball-and-stick representation. (**E**) Functional assay of TolQ mutants, where L142A, T145A, and T178A mutants cause growth defects. All assays were performed in LB medium supplemented with rifampicin (2.5 μg/ml). (**F**) Western blotting results revealing the expression levels of TolQ, TolR, and TolA in the TolQRA mutants. (**G**) Western blot results of the TolQ mutants (L142A, T145A, and T178A) pull-down assay. Similar to the TolQ wild type, all three TolQ mutants could be pulled down by TolR.

TolR forms a dimer via its N-terminal TM domain, with a buried surface area of 455.5 Å^2^. Residues Ile18, Leu21, Leu22, Leu25, Leu26, Leu29, Phe32, and Met33 on TolR contribute to dimer formation (Fig. 4, A to C). The dimeric protomers of TolR are separated from residues Asn17 to Lys13 in the cytoplasm. The TolR dimer adopts a face-to-face arrangement and is positioned within the TolQ pentameric α barrel via its N-terminal TM domain. The α barrel lumen is highly hydrophobic within the IM, transitioning to a negatively charged region in the cytoplasm. This contrasts with β barrel OM proteins, which typically feature hydrophilic lumens. Each TolR protomer occupies the α barrel asymmetrically. TolR_1_ is surrounded by TolQ_1_, Q_2_, and Q_5_, whereas TolR_2_ is flanked by TolQ_3_ and Q_4_ (Fig. 4, D to F, and fig. S5, A to D). Notably, the interaction between TolQ and TolR occurs within the TM domains. The TM domain of TolR_1_ interacts with TM2 and TM3 of TolQ_1_, TolQ_2_, and TolQ_5_, whereas TolR_2_’s TM domain interacts with TM2 and TM3 of TolQ_3_ and TolQ_4_ (Fig. 4, E to F, and fig. S5, A to D). To test whether TolR dimerization and TolR and TolQ interactions are important, TolR mutants of Leu22→Ala (L22A), Leu25→Ala (L25A), Phe32→Ala (F32A), and Met33→Ala (M33A) were generated, and functional assays were performed (Materials and Methods). The TolR mutants of L25A, F32A, and M33A had no effect on cell growth (Fig. 4B). Previous studies, using in vivo cross-linking experiments, have demonstrated that L22 is potentially involved in interactions with TolR (*48*). Structural analysis revealed that this amino acid might participate in the formation of two TolR dimers. Subsequently, we conducted functional experiments to corroborate this observation. The TolR L22A mutant exhibited severe growth defects (Fig. 4, B and C). Our in vivo disulfide-bond formation assay demonstrated that L22C is capable of forming disulfide bonds (Fig. 4C), which further validates and confirms the importance of TolR dimerization for the functionality of the TolQRA machinery.

### The proton transport residues might form proton transport gates

**Fig. 5.**
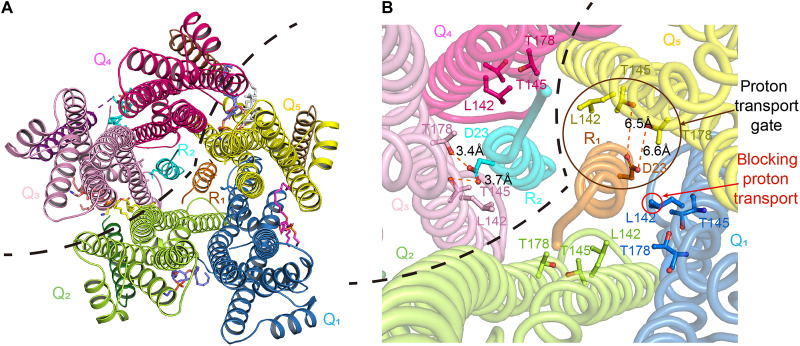
Possible proton transport gates in TolQRA. (**A**) Bottom view of *E. coli* TolQRA at pH 5.4. The TolR_1_ (orange) molecule occupies three TolQ protomers, and TolR_2_ (cyan) takes two protomers of TolQ. (**B**) Possible proton transport gate in TolQRA. Four residues are involved in proton transport: TolQ^L142^ may serve as a gate-blocking residue, whereas TolR^D23^ and TolQ^T145^ function in conjunction with TolQ^T178^ to form proton transport gates. When the distances between TolR^D23^, TolQ^T145^, and TolQ^T178^ are appropriate, the gating residue facilitates proton transport. If the distances become closer or the gating blocking residue is engaged, the proton transport gate closes.

To explore potential proton transport residues of TolR and TolQ, our analysis indicated that the side chains of TolR_2_ Asp23 and TolQ_3_ Thr145/Thr178 (candidate proton transport residues) appear to form hydrogen bonds, with approximately distances of 3.4 and 3.7 Å. In contrast, the side-chain distances between TolR_1_ D23 and TolQ_5_ T145/T178 exceed 6.5 Å (Fig. 5, A and B). These observations may provide preliminary support for the idea that residues D23, T145, and T178 could form candidate proton transport gates, with the putative gate at the TolR_2_ side appearing to adopt a closed conformation, and that at the TolR_1_ side likely in an open conformation (Fig. 5B).

We generated TolQ mutants [Tyr139→Ala (Y139A), Leu142→Ala (L142A), Thr145→Ala (T145A), Leu164→Ala (L164A), and Thr178→Ala (T178A)], and functional assays showed that L142A and T145A caused severe growth defects in the *tolQRA*-deleted strain under rifampicin treatment, whereas T178A induced moderate defects and Y139A/L164A had no effect on cell viability (Fig. 4, E and F). Our pull-down assay results demonstrated that when TolQ was pulled down using TolR, TolQ was detectable in all samples (Fig. 4G). This indicates that these three mutations did not affect the formation of the TolQR complex, which is consistent with the findings of Goemaere *et al.* (*49*). Notably, prior functional studies identified TolQ residues T145, T178, and TolR D23 as critical for proton transport (*49*, *50*), and previous mutagenesis experiments showed that the L142C mutant exhibited sensitivity to colchicine and deoxycholate (*50*), a phenotype consistent with Goemaere *et al.*’s (*49*) findings in D23/T148/T178 mutant analyses, implying that L142, D23, T145, and T178 may collectively participate in proton translocation. Mechanistic parallels exist in other membrane proteins: Voltage-gated proton channel Hv1 uses hydrophobic residues to form a “hydrophobic gasket” that restricts water entry to prevent proton leakage (*51*); the Kv2.1 channel harbors a conserved hydrophobic coupling nexus essential for internal pore gating, a motif shared by Kv/Cav/Nav channel families (*52*); and the proton-coupled folate transporter (PCFT-SLC46A1) requires the hydrophobicity of W299 for substrate transport (*53*). Of note, as a hydrophobic amino acid, TolQ L142 may not directly mediate proton transfer in the same way as the polar residues D23/T148/T178. Instead, it appears to function as a blocking residue, potentially helping to stabilize the conformation of the proton-binding site and mitigate potential membrane leakage (Fig. 5B).

To determine whether ExbBD and MotAB share features similar to those of TolQRA, we analyzed the ExbBD [Protein Data Bank (PDB) ID: 6TYI] and MotAB-FLIG (PDB ID: 8UCS) structures, which revealed that the dimeric ExbD and MotB molecules are asymmetrically located in the pores of ExbB and MotA (fig. S6, A to D), respectively. ExbD_1_ occupies three ExbB protomers, ExbB_1_, ExbB_2_, and ExbB_3_, whereas ExbD_2_ occupies two ExbB protomers, ExbB_4_ and ExbB_5_. The side-chain distances between ExbD_1_ Asp25 (a candidate proton transport residue) and the ExbB_1_ proton transport residues Thr148 (6.8 Å) and Thr181 (5.2 Å) are consistent with an open conformation of the putative proton transport gate. In contrast, ExbD_2_ Asp25 appears to orient toward the opposite side, which may correspond to a closed conformation of this putative gate (fig. S6B). ExbB Leu145 might be a proton transport blocking residue. MotB_1_ occupies MotA_1_, MotA_2_, and MotA_5_, and MotB_2_ occupies MotA_3_ and MotA_4_. The distances between the side chains of the proton transport residues MotB_1_ Asp25 and MotA_5_ Thr164 and Thr192 are 8.9 and 5.5 Å, respectively, which are consistent with a potentially open conformation proton transport gate. In contrast, the proton transport residues MotB_2_ Asp25 and MotA_3_ Thr164 and Thr192 form hydrogen bonds with distances of 2.6 Å, a configuration that may contribute to a closed state of the proton transport gate (fig. S6D). MotA Met161 might be the proton transport blocking residue. These structural analyses suggest the possibility that ExbBD and MotAB may share a similar mechanism involving two proton transport gates, with one appearing to be open and the other likely closed (fig. S6, B and D).

### Conformational changes at different pH values

To investigate the conformational changes of TolQRA under alkaline conditions, we resolved its structure at pH 8.0 to a resolution of 3.6 Å (Materials and Methods). The TolQRA structure at pH 8.0 closely resembles that at pH 5.4, with an RMSD of 0.95 Å across 1237 aligned residues (fig. S7A). At pH 8.0, the TM domain density of all five TolA molecules was more uniform than that of the structure at pH 5.4. TolA_3_ undergoes conformational changes at pH 8.0 compared with its structure at pH 5.4, whereas the other TolA TM domains remain unchanged (fig. S7B). In TolQ, the largest conformational changes occurred in the periplasmic loops and cytoplasmic domains, whereas the TM domains exhibited minimal changes, limited primarily to side chains. Among the TolQ protomers, TolQ_1_ and TolQ_3_ exhibited the most pronounced conformational changes (fig. S7C). The dimeric TolR molecules at pH 8.0 closely align with those at pH 5.4, showing minimal conformational changes, primarily in the side chains.

### Phospholipids binding to TolQRA

The densities located in the grooves between the TolQ protomers on the cytoplasmic side of the IM in the pH 5.4 structure were identified as phosphatidylethanolamine (PE). Despite extraction from the membrane using the detergent *n*-dodecyl-β-d-maltopyranoside (DDM) and purification with LMNG (Materials and Methods), phospholipid molecules remained bound, indicating a tight association between PE molecules and TolQRA (Materials and Methods). The orientation and number of PEs bound vary, which is consistent with the asymmetry of the TolQ protomers in the complex (fig. S8, A to C). Densities were also observed between the TolQ protomer grooves at pH 8.0, although these densities were poorly resolved because of lower resolution. Notably, cryo-EM structures of the ExbBD complex in a nanodisc (PDB ID: 6TYI) and in detergent micelles (PDB ID: 6YE4) revealed four and five phospholipid molecules bound within three and five grooves of the ExbB protomers, respectively, in positions and manners similar to those in TolQRA at pH 5.4 (fig. S8, D to F) (*36*, *54*). The most well-defined PE density was observed between the grooves of TolQ2 and TolQ3 in the TolQRA structure at pH 5.4 (fig. S8, D and G). Residues Arg198, Arg194, Met190, Pro187, and Phe183 of TolQ_2,_ along with residues Phe143, Ile140, Ile136, Thr132, Phe129, His126, Thr125, Thr30, Phe27, Ile23, and Leu22 of TolQ_3,_ contribute to PE binding. Two mutants, mut1 [Ile23→Ala (I23A)/Phe27→Ala (F27A)/Phe129→Ala (F129A)/His126→Ala (H126A)/Thr125→Ala (T125A)] and mut2 [Phe183→Ala (F183A)/Pro187→Ala (P187A)/Met190→Ala (M190A)/Arg194→Ala (R194A)/Arg198→Ala (R198A)], were generated, and functional assays revealed that mut2 affected cell growth (fig. S8B).

### TolQRA dynamics reveal alternative open and closed conformations

To investigate TolQRA machinery dynamics, we analyzed the TolQRA particles at pH 5.4 via the 3D variability display in CryoSPARC (*55*), which enables the capture of distinct conformational changes. TolQ_4_ exhibited relatively low mobility, suggesting that it serves as a structural axis within the complex. TolQ_1_ and TolQ_3_ move in opposite directions, whereas TolQ_2_ and TolQ_5,_ as well asTolR_1_ and TolR_2,_ also move in opposite directions (movies. S1 and S2). TolQ_1_ and Q_3_ exhibit coupled movement in the same direction, as do TolQ_2_ and Q_5_ (fig. S9A). Concurrently, TolA_4_ undergoes notable conformational changes during this process (fig. S9B). Initially, TolR_2_ interacts with TolQ_2_, TolQ_3_, and TolQ_4_ to open the second proton gate for translocation, as observed from the periplasmic perspective. In contrast, TolR_1_ interacts with TolQ_1_ and TolQ_5_, closing the first proton gate to prevent proton transport. As TolQ_2_ and TolQ_5_ shift outward, TolQ_1_ and TolQ_3_ move inward. During this process, TolR_1_ transitions to interact with TolQ_1_, TolQ_2_, and TolQ_5_, closing the second proton transport gate. Subsequently, TolR_1_ interacts with TolQ_1_, Q_2_, and Q_5_ to open the first proton transport gate. A side view revealed that the opening and closing motions of TolQ could facilitate energy transduction to TolA.

## DISCUSSION

**Fig. 6.**
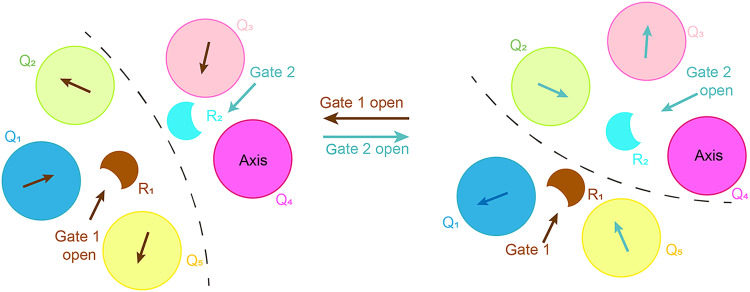
A proposed mechanism by which TolQRA regulates proton transport. There are two distinct proton gates. Gate 1 opens when TolR_1_ interacts with TolQ_1_ (sky blue), TolQ_2_ (limon), and TolQ_5_ (yellow), causing TolQ_1_ and TolQ_3_ to move inward, whereas TolQ_2_ and TolQ_5_ move outward. In contrast, gate 2 opens when TolR_2_ binds to TolQ_2_ (limon), TolQ_3_ (pink), and TolQ_4_ (hot pink), resulting in TolQ_1_ (sky blue) and TolQ_3_ (pink) moving outward and TolQ_2_ (limon) and TolQ_5_ (yellow) moving inward. TolR_1_ (orange) and TolR_2_ (cyan) are also highlighted.

A comparison of the TolQRA structures at pH 5.4 and 8.0 suggested notable conformational changes, apparently concentrated in TolA_3_ and TolR_1_ (fig. S7B). At pH 5.4, the TolQRA structure exhibits apparent unequal positioning of dimeric TolR molecules within the TolQ pentamer, with TolR_1_ appearing to interact with three TolQ protomers and TolR_2_ seemingly engaging with two. Further structural analysis revealed that the three proton transport residues (TolR D23, TolQ T145, and T178) may form a putative open gate at TolR_1_ and a putative closed gate at TolR_2_ (Fig. 5, A and B). To explore the potential proton transport mechanism, 3D variability analysis of TolQRA at pH 5.4 indicated opposing movements between TolQ_1_ and TolQ_3_, TolQ_2_ and TolQ_5_, and TolR_1_ and TolR_2_. These dynamic observations may provide preliminary support for the idea that TolR_1_ could interact with two TolQ protomers, whereas TolR_2_ might engage with three, which could imply that the candidate proton transport residues may form a putative closed gate at TolR_1_ and a putative open gate at TolR_2_. We tentatively propose a potential two-gate mechanism for TolQRA: Gate 1 (associated with TolR_1_ near TolQ) appears to close when TolR_1_ seems to interact with two TolQ protomers. In contrast, gate 2 (associated with TolR_2_) seems to open when TolR_2_ appears to engage three TolQ protomers. The observed conformational changes in TolQRA may contribute to alternating the opening of the two putative proton gates (Fig. 6). Confirmation of the details of proton conduction, however, requires the identification of any “proton wire,” a task that necessitates a higher-resolution structure capable of revealing the location of solvent water molecules.

Superimposition of the TolQR structure at 4.2-Å resolution (PDB ID: 8ODT) onto the TolQRA structure at pH 5.4 revealed conformational changes, with an RMSD of 1.10 Å over 1345 aligned residues. This suggests that some structural rearrangements occur in TolQ_1_, TolQ_2_, TolQ_4_, and TolQ_5_ (fig. S10). In the TolQR structure, TolR_1_ occupies TolQ_1_, TolQ_2_, and TolQ_5_, whereas TolR_2_ occupies TolQ_3_ and TolQ_4_ (fig. S10). Owing to the TolQR structure at low resolution, we are unable to observe the details of the proton transport gates.

Similar systems, such as ExbBD and MotAB, share a 5:2 stoichiometry with TolQRA (*33*, *36*). The ExbBD structure from *E. coli* (PDB ID: 6TYI) aligns with the TolQRA structure at pH 5.4, with an RMSD of 3.85 Å over 1003 aligned residues, whereas the MotAB (PDB ID: 8UCS) structure shows low or no similarity to TolQRA. Despite this, all three systems share key proton transport residues: TolQRA (D23, T145, and T178), ExbBD (D25, T148, and T181) (*36*), MotAB (D25, T164, and T192) (*56*, *57*), and a pentameric α barrel architecture (*31*). ExbBD appears to exhibit unequal occupancy of its pentameric pores, with ExbD_1_ seemingly spanning three ExbB protomers and ExbD_2_ apparently spanning two (PDB ID: 6TYI). This observation could tentatively be consistent with the potential formation of putative open and closed proton transport gates (fig. S6A). In *Clostridium sporogenes* MotAB (PDB ID: 8UCS), MotB_1_ appears to engages three MotA protomers, whereas MotB_2_ seemingly interacts with two, which may be consistence with the possibility that MotAB forms putative proton transport gates analogous to those proposed for TolQRA (fig. S6C). These observations could provide preliminary support for the idea that ExbBD and MotAB may use a potential similar mechanism to TolQRA for mediating proton translocation, potentially via a putative two-gate mechanism.

This study resolves the longstanding debate over TolQRA stoichiometry, demonstrating a 5:2:5 arrangement and providing detailed insights into TolA-TolQ interactions (*4*), as well as proton transport residue functionality. Our findings suggest that the ExbBD-TonB system may also adopt a 5:2:5 stoichiometry, with an arrangement analogous to that of TolQRA.

In a recent parallel study, Yeow *et al.* (*58*) revealed several findings regarding TolQRA in *E. coli*. Consistent with our results, they identified the nonequivalent environments of the two TolR D23 residues. On the basis of distance analysis, they observed that one of the TolR D23 residues is in close proximity to T145 and T178 on the same TolQ partner, suggesting a potential strong interaction. In contrast, the other TolR D23 residue is slightly farther from T145 and T178 on TolQ with a vertical offset, implying a weaker interaction. Yeow *et al.* used these observations to distinguish the distinct protonation states of the two TolR D23 residues, a finding that aligns with the hypothesis of a two-gate proton transport channel proposed in the present study. While their research revealed the TolQRA structure with a stoichiometry of 5:2:1, our work elucidates the TolQRA structure with the stoichiometry of 5:2:5, which suggests that different conformations and stoichiometries of TolQRA might coexist and cooperate in proton-driven force transduction across the bacterial envelope.

In summary, our work provides an important framework for understanding how TolQRA transduces energy from the IM to the OM via the PMF for colicin import, peptidoglycan-OM tethering, cell wall remodeling, and OM phospholipid biogenesis. These findings enhance our understanding of ExbBD and MotAB mechanisms and may inform the development of therapies targeting drug-resistant bacteria.

## MATERIALS AND METHODS

### Expression and purification of TolQRA

The gene fragments encoding TolQRA were amplified by polymerase chain reaction (PCR) using *E. coli* K-12 genomic DNA as a template. These PCR products were then assembled into a modified pTrc99a plasmid containing a small ubiquitin-like modifier tag and an N-terminal eight-histidine tag in TolA, yielding a plasmid named pTrc99a-*tolQRA* (8 × His). *E. coli* C43(DE3) (Weidi) harboring pTrc99a-*tolQRA* (8 × His) was used for protein expression.

The transformed C43(DE3) cells were cultured in Luria broth (LB) supplemented with ampicillin (100 μg ml^−1^) at 37°C until the optical density of the culture reached 0.6 at a wavelength of 600 nm (OD_600_). The proteins were induced by the addition of 0.1 mM isopropyl-β-d-thiogalactopyranoside (IPTG) and incubated for 16 hours at 20°C. The cell pellets were collected and lysed in buffer A [20 mM tris-HCl (pH 8.0) and 300 mM NaCl] using a cell homogenizer at 800 bar (ATS SCIENCE INC), and insoluble cell debris was removed by centrifugation at 12,000*g* for 20 min at 4°C.

The supernatant was subjected to ultracentrifugation at 140,000*g* for 1 hours to collect the membrane fractions. The cell membrane was subsequently solubilized in buffer B [20 mM tris-Cl (pH 8.0), 300 mM NaCl, and 10 mM imidazole] supplemented with 1% (w/v) DDM (Anatrace) at 4°C for 1 hour. Insoluble cell debris was removed by centrifugation at 16000*g* for 30 min at 4°C. The supernatant was collected and loaded onto a 5-ml HisTrap HP column (Cytiva) that had been preequilibrated with buffer B supplemented with 0.01% LMNG (Anatrace). After the column was washed with buffer C [20 mM tris-Cl (pH 8.0), 300 mM NaCl, and 60 mM imidazole] containing 0.01% LMNG (Anatrace), the TolQRA complex was eluted with buffer D [20 mM tris-Cl (pH 8.0), 300 mM NaCl, 300 mM imidazole] containing 0.006% LMNG. The eluted protein was concentrated using centrifugal filters with a 100-kDa molecular weight cutoff (Merck Millipore) and further purified via Superose 6 Increase 10/300GL (Cytiva) in buffer A containing 0.003% LMNG or buffer E [20 mM phosphate-buffered saline and 150 mM NaCl (pH 5.4)] containing 0.003% LMNG to obtain the protein complex under different pH conditions. Last, peak fractions were collected, and protein fractions with the highest purity were concentrated to 6 mg ml^−1^.

### Cryo-EM sample preparation and data collection

A 3-μl drop of the purified sample at a concentration of 6 mg ml^−1^ was applied to a Quantifoil holy carbon grid (R1.2/1.3, 300 mesh Cu). Before sample application, all the grids were glow discharged for 50 s. The grids were subsequently frozen in liquid ethane using a Vitrobot Mark IV. The freezing process involved setting the Vitrobot Mark IV to 8°C and 100% humidity, with no wait time, a blot time of 3 s, and a blot force of +3. Cryo-EM images were collected on a 300-keV Titan Krios (Thermo Fisher Scientific) instrument equipped with a K3 detector (Gatan) and a BioQuantum energy filter. Data were collected in counting mode, with 40 total frames per movie in 3 s, 50 electrons per Å^2^ accumulated dose, and 0.84-Å physical pixel size. The defocus range was set between −1 and −3 μm. For more detailed information about the EM data collection parameters, please refer to table S1.

### Cryo-EM structural determination

The cryo-EM data were processed using CyroSPARC (*55*). The movies were initially preprocessed by CryoSPARC live. Motion correction and contrast transfer function (CTF) estimation were conducted via the Patch Motion Correction and Patch CTF estimation programs.

For TolQRA (pH 5.4), 19,649 micrographs were exported from the CyroSPARC live work session into the CyroSPARC workspace. A total of 20,405,901 particles were automatically picked using Blob Picker. After several rounds of particle 2D classification, the selected 2D class averages were used as templates to pick another round of particles, and five rounds of 2D classification were performed to remove damaged particles and aggregates. The good particles from the Blob Picker and Template Picker were then combined, and duplicated particles were removed, resulting in 470,510 particles subjected to several rounds of ab initio reconstruction, heterogeneous refinement, and nonuniform refinement. Last, nonuniform refinement generated a 3D map with a resolution of 3.18 Å using 122,664 particles.

For TolQRA (pH 8.0), 16,912 micrographs were exported from the CyroSPARC live work session into the CyroSPARC workspace. A total of 12,810,149 particles were automatically picked using Blob Picker. Several rounds of particle 2D classification, ab initio reconstruction, heterogeneous refinement, and reextraction were performed. Last, the selected particles from heterogeneous refinement were combined, followed by particle extraction with a box size of 300 pixels, without any Fourier cropping. The selected 338,644 particles were subjected to ab initio reconstruction as previously described, with the following parameters in the reconstruction: number of ab initio classes = 3, initial resolution = 20 Å, and maximum resolution = 5 Å. Heterogeneous refinement was then performed. The best class of 170,384 particles was selected for ab initio reconstruction and heterogeneous refinement, leaving 160,613 particles for nonuniform refinement, which yielded a 3.60-Å map on the basis of Fourier shell correlation.

### Cryo-EM model building and refinement

To construct the structures of TolQRA, the initial main-chain model was generated using the DeepTracer server (https://deeptracer.uw.edu), with the corresponding cryo-EM map as input. The predicted models from AlphaFold2 (Phenix Colab) were divided into multiple fragments, which were then docked into the cryo-EM maps using ChimeraX, which is based on the DeepTracer main chain model (*59*). For TolQRA structures at pH 5.4 and pH 8.0, subsequent adjustments of the model fragments were performed with Coot and ChimeraX to better fit the cryo-EM maps (*60*). Last, real-space refinement was conducted in Phenix for further optimization (*61*). All representations of densities and models were generated by ChimeraX or PyMOL (*62*).

### Cryo-EM 3D variability analysis

To investigate the conformational changes of the TolQRA complex, 3D Variability Analysis from CryoSPARC was used. For TolQRA (pH 5.4), the final 160,613 particles used in the final map generation were put into the 3D Variability Analysis to generate three modes at a resolution filter of 3.5 Å together with a mask covering the whole complex. Then, 3D Variability Display (simple mode) was used to generate the volume series, yielding three volume sets and 20 maps in each set. To generate the volume movies, the middle 16 maps of the total 20 maps of each set were imported into Chimera X as the volume series, and the movie was generated in oscillation mode and recorded at a volume level of 0.12 to 0.14 with dust hidden. After the outputs and movies are checked, one of the volume sets generates a continuous and reasonable volume series displaying the complex conformational changes inside the TolQRA particle stacks.

### Site mutagenesis and functional assays

An *E. coli* K12 chromosomal *tolQRA* deletion strain was constructed using a modified pCas/pTargetF system (guide RNA sequence, TGGTATTGGTCAGTACACCG). Positive clones were confirmed by PCR (PCR primer, seq-F: 5′-TGCCGATATTGTTCTCATTGTCGGG-3′; seq-R: 5′-AGCCACTGCTTGTTCACTTTGTACG-3′) (*63*). The Δ*tolQRA* strain was then constructed and named WYD1 (MG1655 ∆*tolQRA*).

All single or double mutations were generated following the site-directed mutagenesis protocol of Liu and Naismith (*64*). All the mutations were amplified from pTrc99a-*tolQRA*, a Flag tag (DYKDDDDK) on the C terminus of TolQ, an Myc tag (EQKLISEEDL) was added to the C terminus of TolR, and a strep tag was added to the C terminus of TolA. These mutants were subsequently transformed into the WYD1 strain. A single colony from each transformation was inoculated into 10 ml of LB medium supplemented with ampicillin (100 μg/ml) at 37°C overnight. The cell pellets were collected and diluted to an OD_600_ of 0.4. Tenfold serial dilution viability assays were carried out and dripped onto LB agar plates containing rifampicin (2.5 μg/ml). Cell growth was recorded after overnight culture at 37°C. All the assays were performed at least three times.

### Western blotting

The protein expression levels of pTrc99a-*tolQ*(*Flag*)*R*(*Myc*)*A*(*Strep*) and its mutants were determined by Western blotting. The WYD1 strains cultured overnight with TolQRA, TolQRA mutants, or empty plasmids were inoculated into 10 ml of LB supplemented with ampicillin (100 μg ml^−1^) and 0.1 mM IPTG. After incubation at 37°C for 6 hours, the cells were harvested via centrifugation at 6000*g* and 4°C for 15 min. The cell pellets were then resuspended in 0.5 ml of buffer A and lysed via sonication for 5 min on ice. The insoluble cell debris was removed by centrifugation at 12,000*g* for 20 min at 4°C. The supernatant was mixed with 5 × SDS–polyacrylamide gel electrophoresis (SDS-PAGE) loading buffer.

A 20-μl sample was loaded onto a 4 to 12% SDS-PAGE gel for 50 min. The proteins were then transferred to a polyvinylidene difluoride membrane, washed with tris-buffered saline (TBS) buffer, and blocked in TBS buffer supplemented with 5% skim milk at 4°C overnight. The membranes were then incubated with anti-Flag (1:1000 dilution) (Sigma-Aldrich, catalog no: F1804), anti-Myc (1:5000 dilution) (Sigma-Aldrich, catalog no: 4439), or anti-Strep monoclonal antibody (1:5000 dilution) at room temperature for 2 hours. After incubation, the membranes were washed with TBST [20 mM tris-HCl (pH 8), 300 mM NaCl, and 0.1% Tween-20] three times and then incubated with goat anti-mouse immunoglobulin G antibody (1:4000 dilution) for 1 hour. After washing with TBST three times, the membranes were finally incubated with enhanced chemiluminescence substrate before imaging. The images were acquired with a Bio-Rad ChemiDoc Imaging System. All the experiments were repeated at least three times.

### Pull-down assay

Using the pTrc99a-*tolQ*(*Flag*)*R*(*Myc*)*A*(*Strep*), pTrc99a-*tolQ^L142A^*(*Flag*)*R*(*Myc*)*A*(*Strep*), pTrc99a-*tolQ^T145A^*(*Flag*)*R*(*Myc*)*A*(*Strep*), and pTrc99a-*tolQ^T178A^*(*Flag*)*R*(*Myc*)*A*(*Strep*) plasmids as templates, an 8×His tag was introduced downstream of the Myc tag to detect the interaction between TolQ and TolR. Concurrently, the previously constructed pTrc99a-*tolQ* (*Flag*)*R*(*Myc*)*A^H22A^*(*Strep*) plasmid was used to assess the interaction between TolA and TolQ. All plasmids were transformed into *E. coli* BL21(DE3) (Weidi) and cultured at 37°C. When the OD_600_ reached 0.6 to 0.8, protein expression was induced by adding 0.1 mM IPTG, followed by incubation at 20°C for 6 hours. Cells were harvested and lysed in buffer A. Cells expressing the five plasmids (pTrc99a-*tolQ*(*Flag*)*R*(*Myc&his*)*A*(*Strep*), pTrc99a-*tolQ^L142A^*(*Flag*)*R*(*Myc&his*)*A*(*Strep*), pTrc99a-*tolQ^T145A^*(*Flag*)*R*(*Myc&his*)*A*(*Strep*), pTrc99a-*tolQ^T178A^*(*Flag*)*R*(*Myc&his*)*A*(*Strep*), and pTrc99a) were purified using a Ni–nitrilotriacetic acid column (Cytiva). Eluates were analyzed by 4 to 20% SDS-PAGE and subjected to Western blot analysis using anti-Flag and anti-Myc tag antibodies. Cells expressing the three plasmids [pTrc99a-*tolQ* (*Flag*)*R*(*Myc*)*A^H22A^* (*Strep*), pTrc99a-*tolQ* (*Flag*)*R*(*Myc*)*A*(*Strep*), and pTrc99a] were purified using a 1-ml Strep column (Smart-Lifescience). Eluates were analyzed by 4 to 20% SDS-PAGE and subjected to Western blot analysis with anti-Flag and anti-Strep tag antibodies. All the experiments were repeated at least three times.

### In vivo disulfide-bond formation assay

Plasmid pTrc99a-*tolQ*(*Flag*)*R*(*Myc*)*A*(*Strep*) was used as the template to introduce L22C and F32C mutations, with an 8×His tag inserted downstream of the Myc tag. The resulting plasmid was transformed into the WYD1 strain. Strains harboring the target plasmid were inoculated into 10 ml of LB medium supplemented with ampicillin and cultured at 37°C. When the OD_600_ reached 0.6 to 0.8, protein expression was induced by adding 0.1 mM IPTG, followed by incubation at 20°C for 6 hours before harvesting. Cell pellets were resuspended in 1 ml of ice-cold buffer A and incubated with 4,4′-dipyridyl disulfide (final concentration 0.4 mM) on ice for 30 min. Cells were then lysed by sonication and centrifuged at 12,000*g* for 20 min at 4°C to remove insoluble cellular debris. The supernatant was purified using a nickel affinity chromatography column. Eluates were mixed with 5× SDS-PAGE loading buffer (with and without dithiothreitol) and analyzed by 4 to 20% SDS-PAGE, followed by Western blot analysis using an anti-Myc tag antibody.
