# Supplementary material for: Structural basis of outer membrane biogenesis and cell division by Tol/Pal nanomachinery
Source: Sci Adv. 2026 Mar 6;12(10):eadw6719. doi: 10.1126/sciadv.adw6719 (PMC12965293; doi:10.1126/sciadv.adw6719)
Supplement: Supplementary file 1 — Figs. S1 to S10 Table S1 Legends for movies S1 and S2 [file sciadv.adw6719_sm.pdf]

Science Advances
AAAS

Supplementary Materials for

Structural basis of outer membrane biogenesis and cell division by Tol/Pal nanomachinery

Yatian Chen *et al.*

Corresponding author: Zhengyu Zhang, zhengyu.zhang@whu.edu.cn;
Changjiang Dong, changjiangdong@whu.edu.cn

Sci. Adv. **12**, eadw6719 (2026)
DOI: 10.1126/sciadv.adw6719

The PDF file includes:

Figs. S1 to S10
Table S1
Legends for movies S1 and S2

Other Supplementary Material for this manuscript includes the following:

Movies S1 and S2

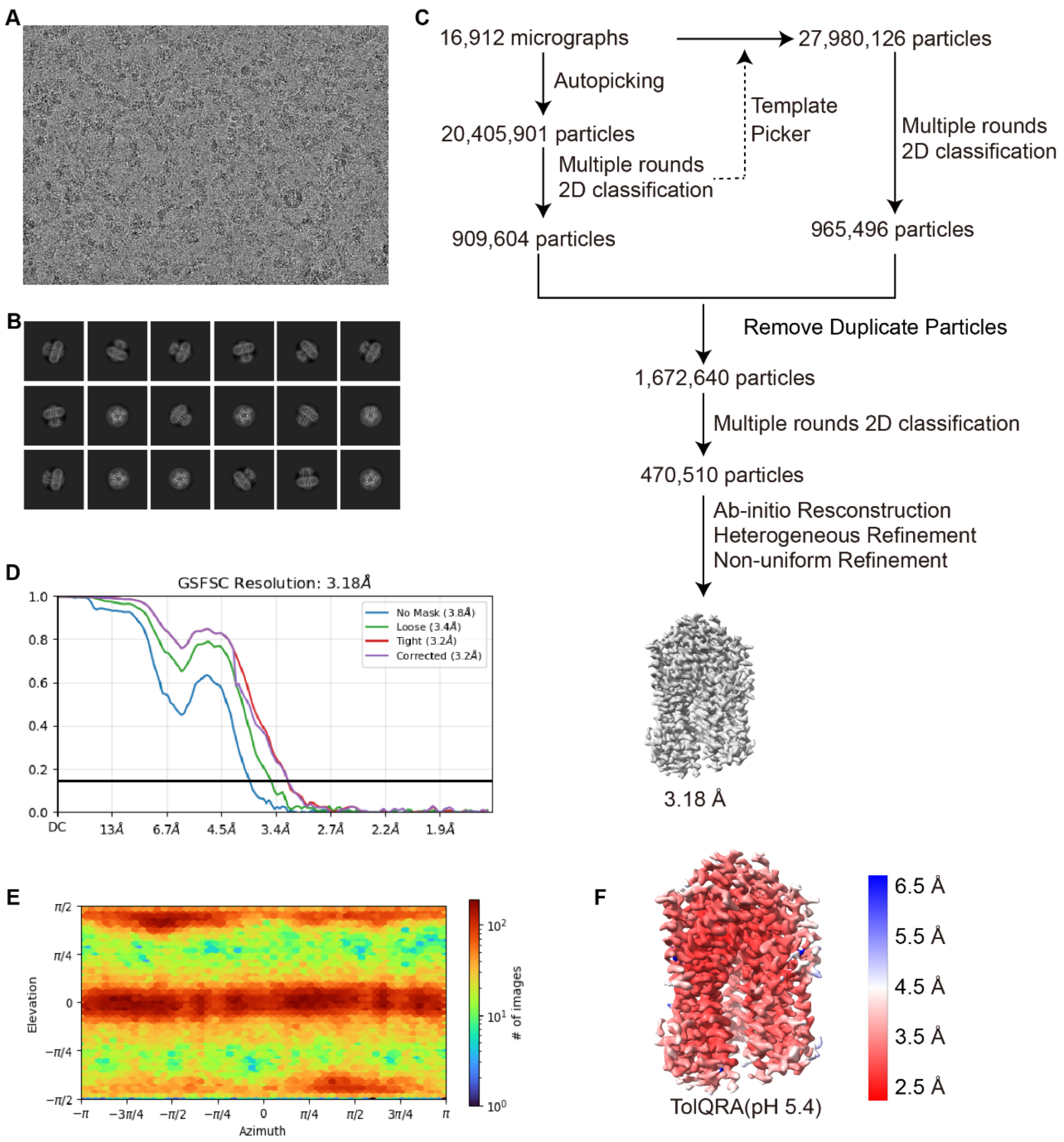

Fig. S1. Data collection of TolQRA (pH 5.4) and simplified workflow of data processing. (**A**) A representative cryo-EM micrograph. (**B**) Representative 2D classification classes of TolQRA (pH 5.4). (**C**) A simplified flowchart of cryo-EM data processing. (**D**) Gold-standard FSC curves of the final cryo-EM maps of TolQRA (pH 5.4) from CryoSPARC. (**E**) Direction distribution iteration. (**F**) The overall cryo-EM maps of TolQRA (pH 5.4) are colored according to the local resolution.

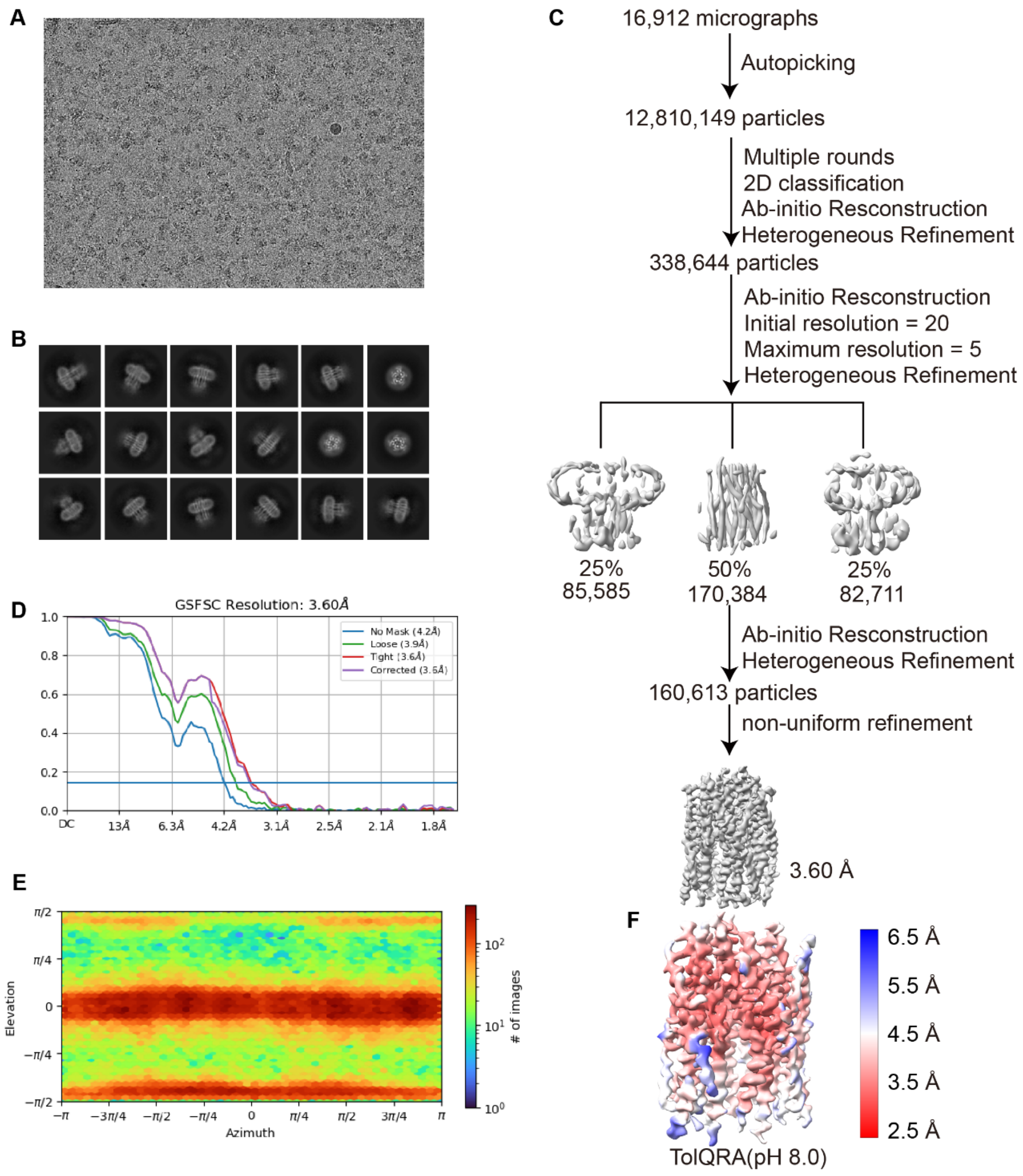

Fig. S2. Data collection of TolQRA (pH 8.0) and simplified workflow of data processing. (**A**) A representative cryo-EM micrograph. (**B**) Representative 2D classification classes of TolQRA (pH 8.0). (**C**) A simplified flowchart of cryo-EM data processing. (**D**) Gold-standard FSC curves of the final cryo-EM maps of TolQRA (pH 8.0) from CryoSPARC. (**E**) Direction distribution iteration. (**F**) The overall cryo-EM maps of TolQRA (pH 8.0) are colored according to the local resolution.

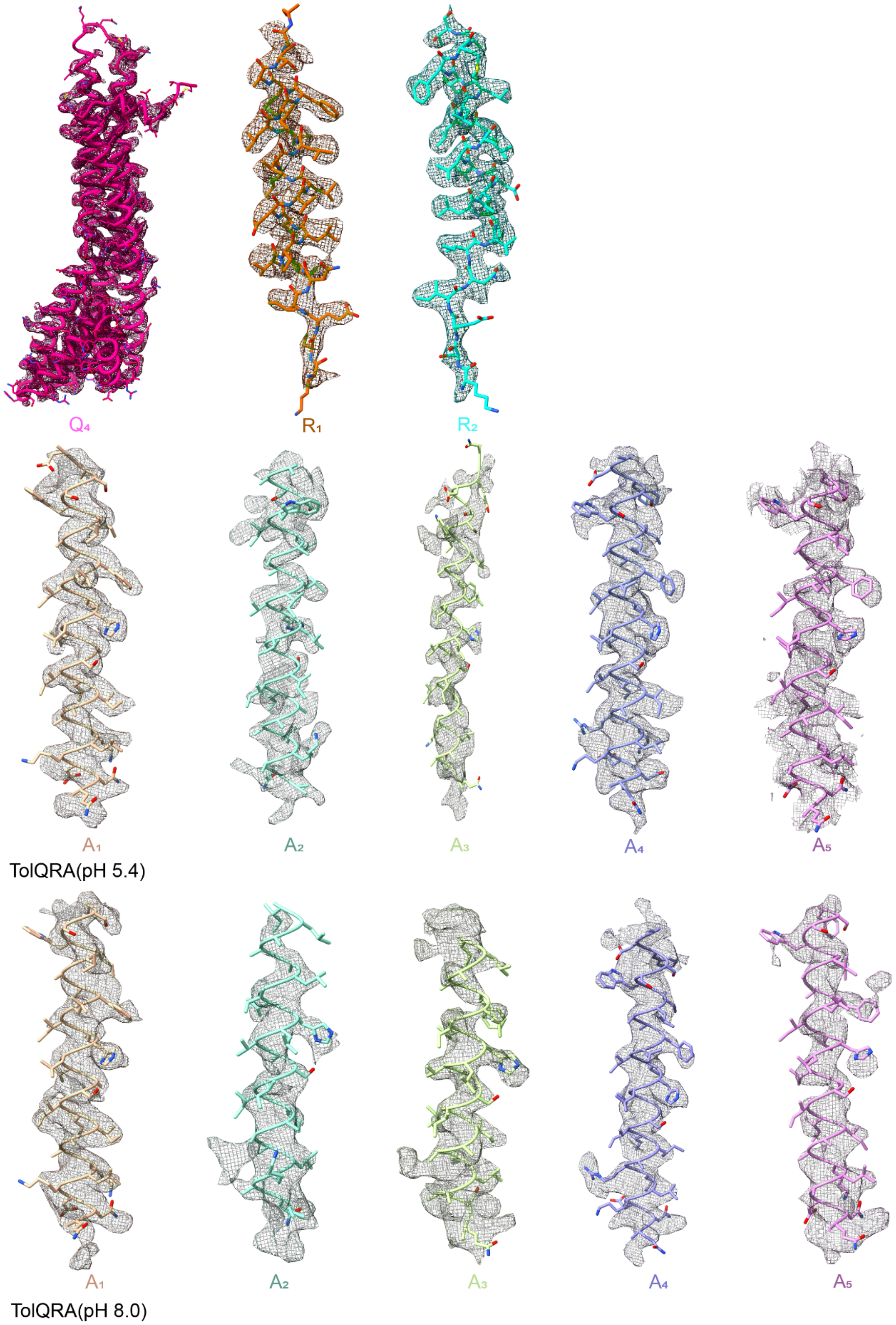

Fig. S3. Partial cryo-EM map of TolQRA in this study.

Cryo-EM maps of $TolQ_4$, $TolR_1$, $TolR_2$, $TolA_1$, $TolA_2$, $TolA_3$, $TolA_4$, $TolA_5$ in TolQRA (pH 5.4), and $TolA_1$, $TolA_2$, $TolA_3$, $TolA_4$, $TolA_5$ in TolQRA (pH 8.0).

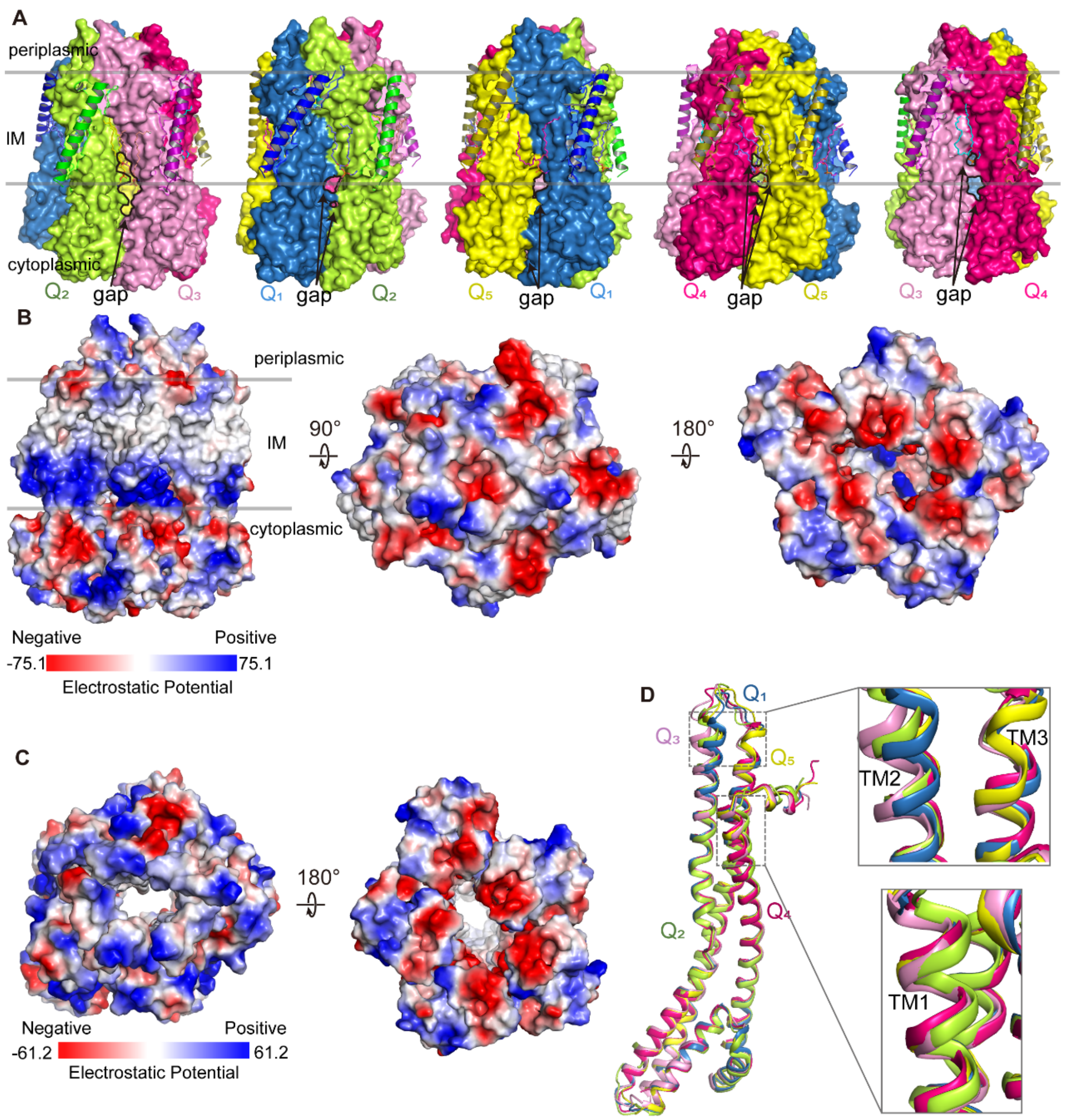

Fig. S4. Surface area analysis, electrostatic analysis and TolQ superimposition of TolQRA (pH 5.4). (**A**) Buried surface areas between protomers Q_1 and Q_2, Q_2 and Q_3, Q_3 and Q_4, Q_4 and Q_5, and Q_5 and Q_1. The gaps between two copies of TolQ are labelled. The gap between Q_2 and Q_3 is the largest, while the gap between Q_4 and Q_5 is the narrowest. $TolQ_1$, $TolQ_2$, $TolQ_3$, $TolQ_4$ and $TolQ_5$ are colored sky blue, limon, pink, hot pink and yellow, respectively. (**B**) Electrostatic analysis of TolQRA (pH 5.4), viewed from the side, top and bottom. The red color indicates the negatively charged area, and the blue color indicates the positively charged area. (**C**) Electrostatic analysis of the TolQ gate, viewed from the top and bottom. (**D**) The superimposition of five copies of TolQ in TolQRA (pH 5.4). The main conformational changes occur in TM1, 2 and 3.

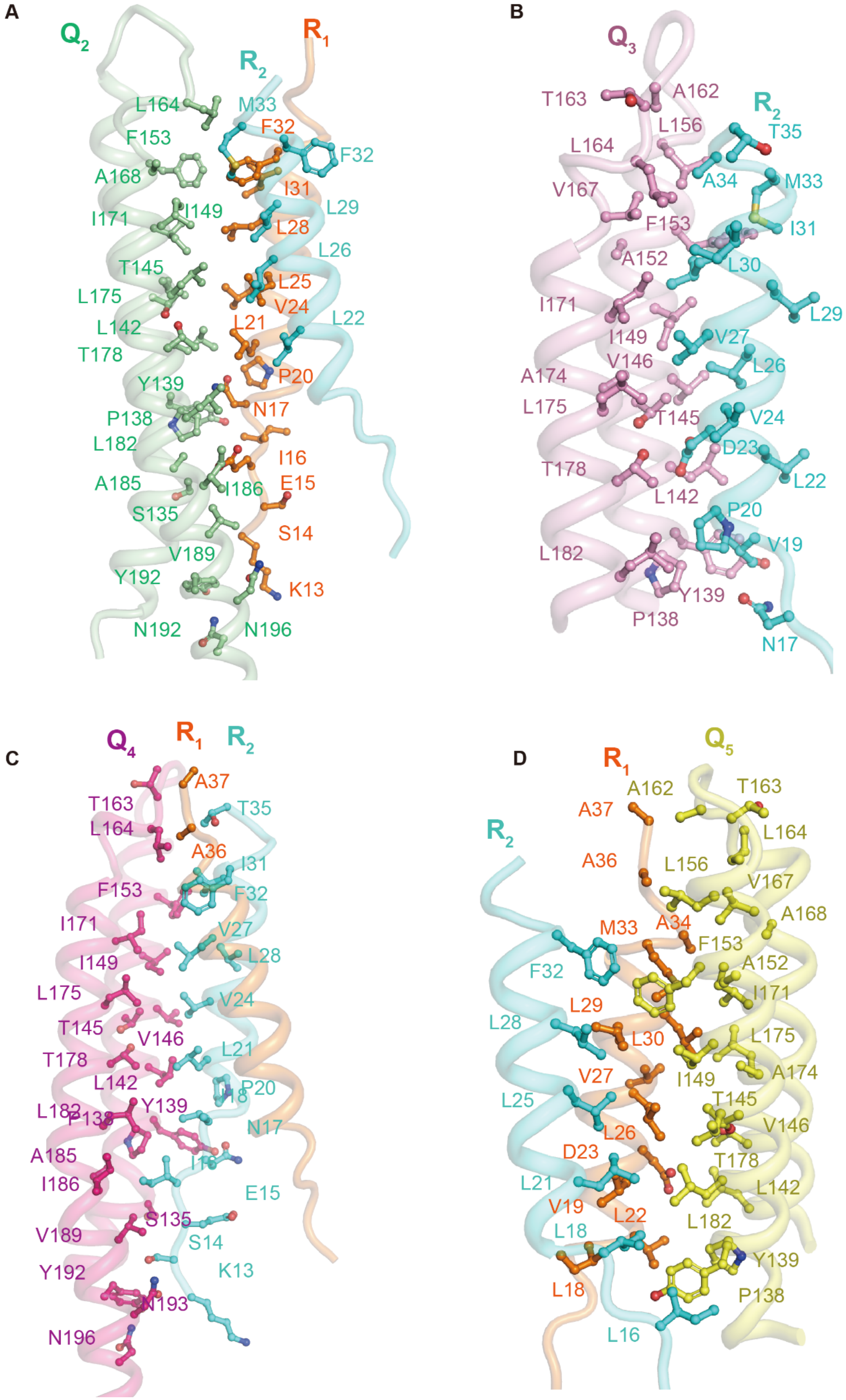

A
Q2
R2
R1
L164
M33
F32
F153
F32
A168
I31
I171
I149
L29
L28
T145
L26
L175
L25
V24
L142
L21
L22
T178
P20
Y139
N17
P138
L182
I16
A185
I186
E15
S135
S14
V189
Y192
K13
N192
N196
B
Q3
T163
A162
R2
L156
T35
L164
A34
M33
V167
F153
I31
A152
L30
I171
L29
I149
V27
V146
L26
A174
L175
T145
V24
D23
T178
L22
L142
P20
L182
V19
Y139
P138
N17
C
Q4
R1
R2
A37
T163
T35
L164
A36
I31
F153
F32
I171
V27
I149
L28
L175
V24
T145
V146
T178
L21
L142
P20
N17
L182
Y139
A185
I186
E15
S135
V189
S14
Y192
K13
N193
N196
D
R1
Q5
A162
T163
A37
R2
L164
A36
L156
V167
A168
M33
A34
F153
F32
A152
I171
L29
L28
L30
L175
V27
I149
A174
L25
T145
L26
V146
D23
T178
L21
L142
V19
L22
L182
L18
Y139
L18
P138
L16

Fig. S5. Interaction between TolQ and TolR. (**A**) $TolR_1$ interacts with $TolQ_2$ and $TolR_2$. (**B**) $TolR_2$ interacts with $TolQ_3$. (**C**) $TolR_2$ interacts with $TolQ_4$ and $TolR_1$. (**D**) $TolR_1$ interacts with $TolR_2$ and $TolQ_5$. $TolQ_1$, $TolQ_2$, $TolQ_3$, $TolQ_4$ and $TolQ_5$ are colored sky blue, limon, pink, hot pink and yellow, respectively. $TolR_1$ and $TolR_2$ are colored in orange and cyan, respectively. The side chains are shown as balls and sticks.

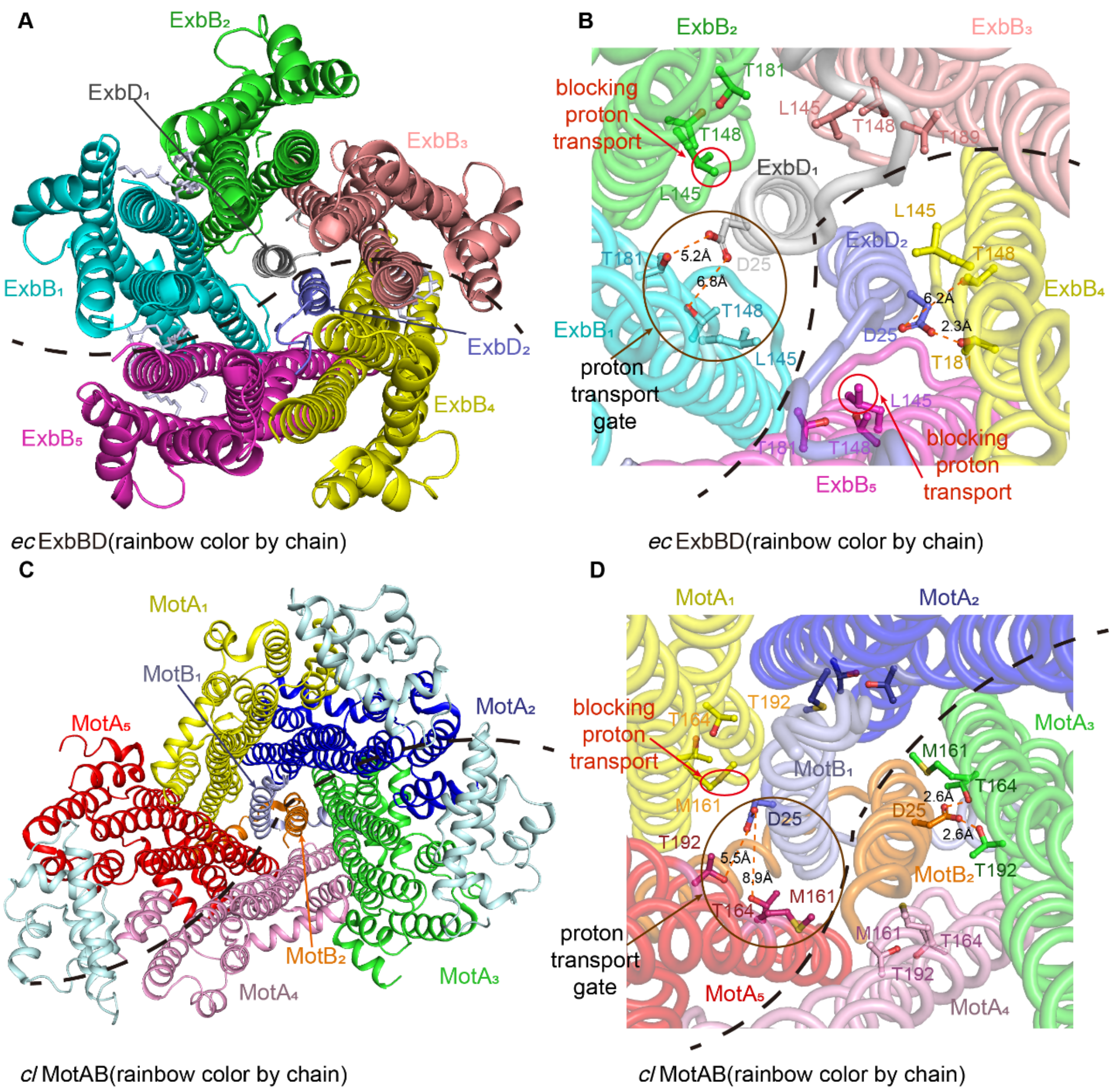

Fig. S6. The cryo-EM structure of *E. coli* ExbBD and *C. sporogenes* MotAB-FLIG. (**A**) Bottom view of *E. coli* ExbBD (*ec*ExbBD) (PDB ID: 6TYI). The $ExbD_1$ molecule occupies three ExbB protomers, and $ExbD_2$ takes two protomers of ExbB. (**B**) Possible proton transport gate in *ec*ExbBD. Four residues are involved in proton transport: $ExbB^{L145}$ may serve as a gate-blocking residue, whereas $ExbD^{D25}$ and $ExbB^{T148}$ function in conjunction with $ExbB^{T181}$ to form proton transport gates. When the distances between $ExbD^{D25}$, $ExbB^{T148}$, and $ExbB^{T181}$ are appropriate, the gating residue facilitates proton transport. If the distances become closer or the potential gate blocking residue is involved, the proton transport gate is closed. (**C**) Bottom view of *C. sporogenes* MotAB-FLIG (*cl*MotAB) (PDB ID: 8UCS), in which $MotB_1$ occupies three MotA protomers and $MotB_2$ occupies two MotA protomers. (**D**) Possible proton transport gate in *cl*MotAB. Four residues are involved in proton transport: $MotA^{M161}$

may serve as a gate-blocking residue, whereas $MotB^{D25,}$ $MotA^{T164}$ and $MotA^{T192}$ might form proton transport gates. When the distances between $MotB^{D25}$, $MotA^{T164}$, and $MotA^{T192}$ are appropriate, the gating residue facilitates proton transport. If the distances become closer or the potential gate blocking residue is approached, the proton transport gate is shut down.

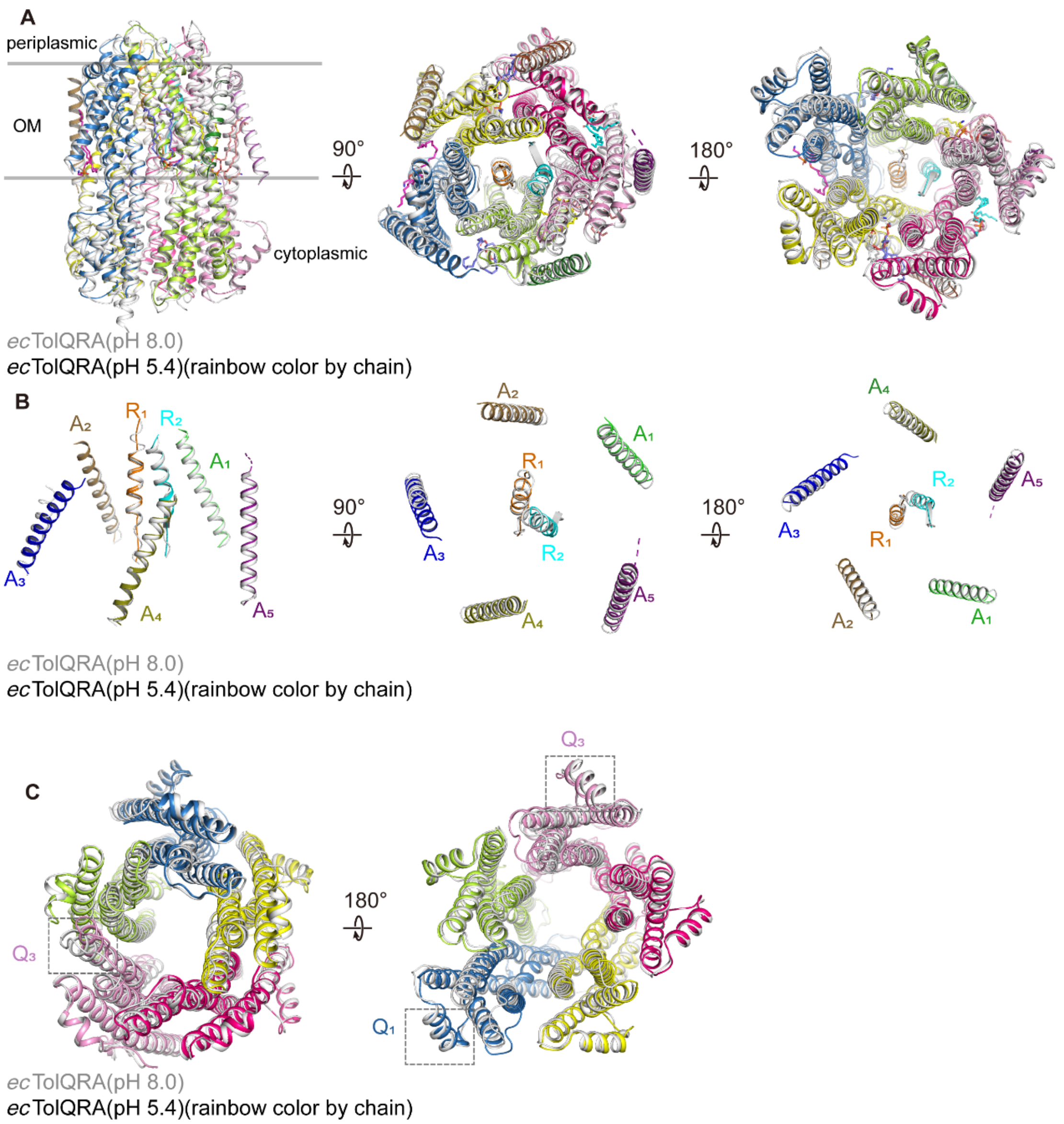

Fig. S7. The superimposition of TolQRA (pH 5.4) and TolQRA (pH 8.0). (**A**) The overall structure of TolQRA (pH 8.0) superimposed on TolQRA (pH 5.4), viewed from the side, top and bottom. These two structures are similar, with an RMSD of 0.95 Å over 1237 aligned residues. *ec*TolQRA (pH 8.0) is colored gray, and *ec*TolQRA (pH 5.4) is rainbow color by chain. (**B**) Superimposition of TolA in TolQRA (pH 8.0) and TolQRA (pH 5.4). $TolA_3$ undergoes the largest conformational changes, whereas the other four TolAs are similar. ToA_1, $TolA_2$, $TolA_3$, $TolA_4$ and $TolA_5$ are colored in deep olive, olive, blue, green and purple. $TolR_1$ and $TolR_2$ are colored in orange and cyan, respectively. (**C**) Conformational changes occur in $TolQ_1$ and $TolQ_3$ during the superimposition of TolQRA (pH 8.0) and TolQRA (pH 5.4). $TolQ_1$, $TolQ_2$, $TolQ_3$, $TolQ_4$ and $TolQ_5$ are colored sky blue, limon, pink, hot pink and yellow, respectively.

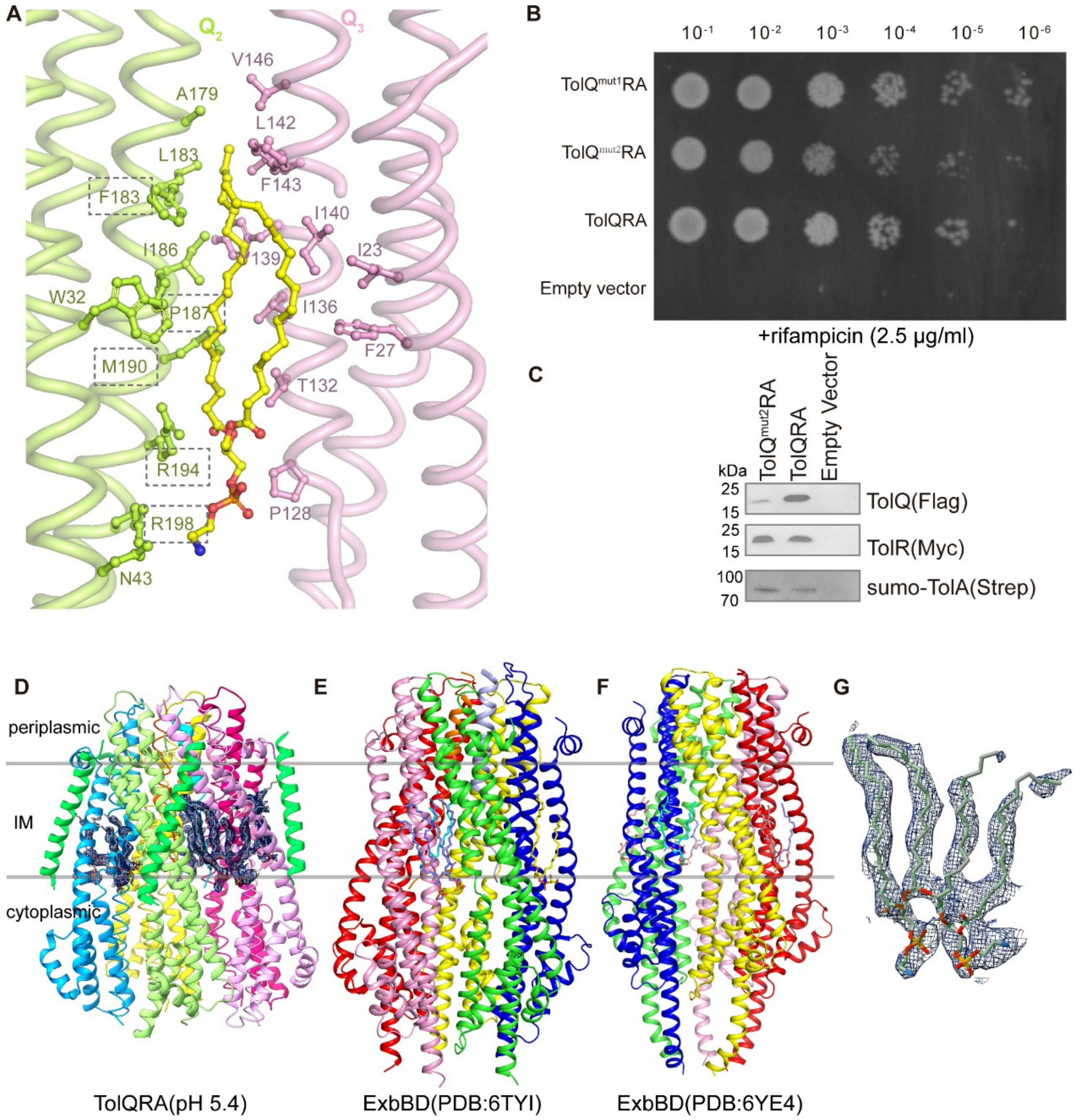

Fig. S8. TolQ residues involved in the TolQ–PE interaction. (**A**) Residues that interact with TolQ and PE. $TolQ_2$ and $TolQ_3$ are colored limon and pink, respectively. The side chains are shown as balls and sticks. (**B**) Functional assay of TolR mutants. All assays were performed in LB medium supplemented with rifampicin (2.5 µg/ml). (**C**) The results of the supernatant Western blotting revealed the expression levels of TolQ, TolR and TolA mutants. (**D**) PE-bound TolQRA (pH 5.4) is rainbow color by chain. (**E**) PE-bound ExbBD (PDB ID: 6TYI) is rainbow color by chain. (**F**) PE-bound ExbBD (PDB ID: 6YE4). (**G**) The PE bound to TolQRA (pH 4.5) is rainbow color by chain.

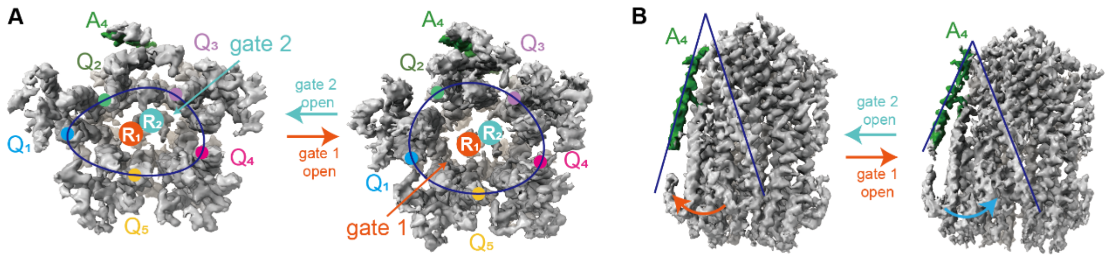

Fig. S9. The cryosparc 3D variability analysis of TolQRA (pH 5.4) revealed conformational changes. (**A**) $TolQ_1$ and $TolQ_3$ move in the same direction, whereas $TolQ_2$ and $TolQ_5$ move in the same direction. $TolQ_1$, $TolQ_2$, $TolQ_3$, $TolQ_4$ and $TolQ_5$ are colored sky blue, limon, pink, hot pink and yellow, respectively. $TolR_1$ and $TolR_2$ are colored in orange and cyan, respectively. (**B**) $TolA_4$ exhibits distinct conformational changes in two states. $TolA_4$ is colored in green.

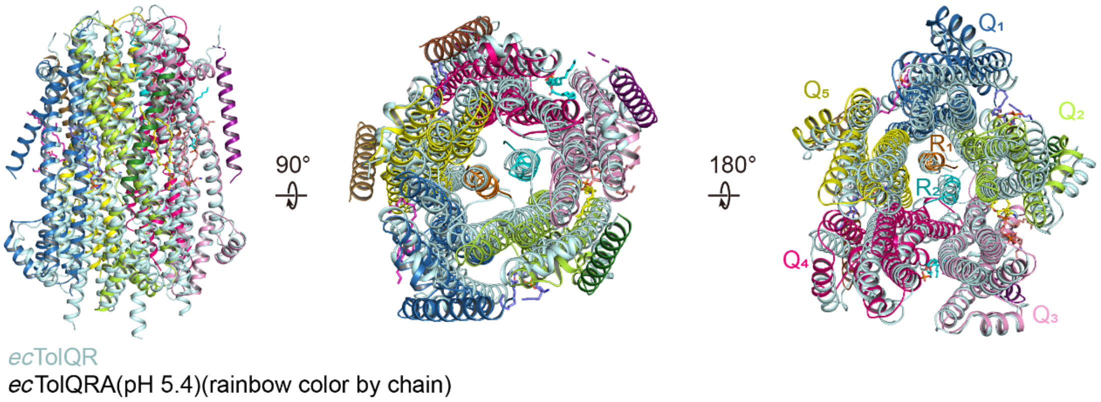

Fig. S10. The superimposition of *E. coli* TolQR and *E. coli* TolQRA (pH 5.4). The superimposition of *E. coli* TolQR (*ec*TolQR) (PDB ID: 8ODT) and *E. coli* TolQRA (*ec*TolQRA) (pH 5.4), viewed from the side, top and bottom. Conformational changes occur in TolQR, with an RMSD of 1.10 Å over 1345 aligned residues. *ec*TolQR is colored in shadow green, and *ec*TolQRA (pH 5.4) is rainbow color by chain.

Table S1. Cryo-EM structure determination parameters and model statistics.

	TolQRA (pH 5.4)<br>PDB ID:9KPZ<br>EMDB ID:62492	TolQRA (pH 8.0)<br>PDB ID:9KQ0<br>EMDB ID:62493
**Data collection and processing**		
Microscope	Titan krios	Titan krios
detector	K3	K3
Magnification	105,000	105,000
Voltage (KV)	300	300
Electron exposure (e^{-}/Å^{2})	50	50
Defocus range (μm)	-1 to -3	-1 to -3
Pixel size (Å)	0.84	0.84
Symmetry imposed	C1	C1
Initial particle images (no.)	20,405,90	112,810,149
Final particle images(no.)	470,510	160,613
Map resolution (Å)	3.18	3.60
FSC threshold	0.143	0.143
**Refinement**		
Map sharpening B factor (Å)	-99.8	-116.2
Model composition		
Nonhydrogen atoms	10466	10087
Protein residues	1297	1290
Ligands	7	0
B-factors (Å)		
Protein	73.01	102.03
Ligand	58.72	--
R.M.S deviations		
Bond length (Å)	0.003	0.002
Bond angles (°)	0.590	0.480
Validation		
Molprobity score	1.65	1.50
Clashscore	7.55	6.53
Ramachandran plot		
Favoured (%)	96.38	97.23
Allowed (%)	3.46	2.69
Outliers (%)	0.16	0.08

Movie. S1. The 3D variability analysis of the bottom view of TolQRA (pH 5.4) in CryoSPARC.

Movie. S2. The 3D variability analysis of the side view of TolQRA (pH 5.4) in CryoSPARC.
